# MiR-520a-5p/PPP5C regulation pattern is identified as the key to gemcitabine resistance in pancreatic cancer

**DOI:** 10.3389/fonc.2022.903484

**Published:** 2022-07-25

**Authors:** Ruibiao Fu, Qian Shao, Bin Yang, Yan Chen, Qinghuang Ye, Xi Chen, Jinhui Zhu

**Affiliations:** Department of Hepato-Pancreato-Biliary (HPB) Surgery, Second Affiliated Hospital, Zhejiang University School of Medicine, Hangzhou, China

**Keywords:** Pancreatic cancer, PPP5C, miR-520-5p, gemcitabine, autophagy

## Abstract

**Objective:**

To explore the effects of the expression level of miR-520-5p/PPP5C in pancreatic cancer cells and exosomes on cell viability, angiogenesis, autophagy, which involved in the mechanism of gemcitabine resistance in pancreatic cancer.

**Methods:**

APSC-1 cell line was treated with gemcitabine, after which its exosomes were extracted for NTA assay. Subsequently, the drug resistance of APSC-1 cells was assayed using CCK8, as well as the activity of HUVEC cells treated with exosomes from each group of APSC-1 cells after drug resistance treatment as well as overexpression treatment. Five groups of HUVEC cells treated with exosomes were subjected to *in vitro* tubule formation assay. levels of PPP5C in each group of ASPC-1 cells and their exosomes, levels of overexpressed PPP5C, and related exosomal proteins were examined by WB. mRNA expression levels of PPP5C and levels of miR-520a were examined by qPCR The relationship between miR-520a-5p and PPP5C was investigated. After that, the autophagy of PPP5C was detected. Finally, it was analyzed by TCGA database for survival prognosis analysis.

**Results:**

APSC-1 cells had an IC50 value of 227.1 μM for gemcitabine, elevated PPP5C expression, drug resistance, and enhanced HUVEC cell activity; exosomes CD9, CD63, and CD81 were significantly expressed in all groups; meanwhile, enhanced PPP5C expression not only promoted *in vitro* tubule formation but also increased autophagy levels; meanwhile, its relationship with miR-520-5p and There was a targeted inhibitory relationship between its level and miR-520-5p and PPP5C, and its elevated level also led to a decrease in the survival level of patients over 3-5 years.

**Conclusion:**

PPP5C has a prognostic role in pancreatic cancer by promoting the value-added and invasion of pancreatic cancer cells, and a targeted inhibitory relationship between miR-520-5p and PPP5C was found.

## 1 Introduction

Pancreatic cancer is one of the most lethal human tumors with a poor prognosis and a five-year survival rate of less than 10% (1). The incidence and mortality of this disease is predicted to increase dramatically worldwide by 2040 (2). The extreme lack of treatment options, the frequent occurrence of chemotherapy resistance, and its metastatic nature make the prognosis of pancreatic cancer clinical treatment very poor (3–6). And since the occurrence of PPP5C is closely related to the abnormal activation of tumor driver genes, the exploration of pancreatic cancer-related genes has been of increasing interest to researchers in recent years (7).

Through genome-wide association studies, increasing evidence has identified significant associations between pancreatic cancer susceptibility, variants in relevant genes (at least 23 genome-wide significant susceptibility loci), and pancreatic cancer risk (8, 9). However, because of the small sample size of genome-wide association studies related to pancreatic cancer, compared with, for example, breast and colorectal cancer, there are fewer associated genes and fewer variant sites in pancreatic cancer (8). One of these, a member of the phosphoprotein phosphatase (PPP) family of Ser/Thr phosphatases, Ser/Thr protein phosphatase 5 (PPP5C), is encoded by the PPP5C gene (10). Substrates of PPP5C include the glucocorticoid receptor (GR), the tumor suppressor p53, Hsp90, and the co-chaperone Cdc37 (11). Because of this property, PPP5C has been linked to asthma, cardiac contractility and heart failure, diabetes, lipid metabolism, and obesity (12–14). On the cancer side, the role of PPP5C in proliferation and cell survival and its unique structure make it a potentially attractive therapeutic target, and elevated PPP5C expression has been found to increase proliferation in most cells in breast and renal cancer clinical studies (15). The role of PPP5C in pancreatic cancer is similar to that in other cancers: it leads to the progression of pancreatic cancer by enabling tumors to develop resistance to gemcitabine (16). Therefore, targeting the inhibition of PPP5C is particularly important in the treatment of pancreatic cancer, while miRNAs regulate the properties of mRNA expression by binding to its 3’-UTR (17, 18). A type of miRNA, miR-520a-5p in NSCLC, can reverse cancer progression in a near step through enrichment, and a study showed that miR-520a-5p is a tumor suppressor in NSCLC and plays an important role in angiogenesis (19). However, the role of this type of miRNA in pancreatic cancer, as well as its relationship with PPP5C, has been less well-studied.

This study will investigate the expression levels of PPP5C in cells as well as exosomes and the effect of its expression levels on gemcitabine resistance, cellular activity, angiogenesis, and cellular autophagy levels in pancreatic cancer, analyze its targeting relationship with miR-520-5p, and obtain the effect of PPP5C on survival over 3-5 years by bioinformatics analysis.

## 2 Materials and methods

### 2.1 Methods

#### 2.1.1 Construction of gemcitabine-resistant APSC-1 cell line

APSC-1 cells were treated with different doses of gemcitabine: 0 μM, 0.05 μM, 0.5 μM, 5 μM, 50 μM and 500 μM for 48 h. After these treatments, the cell lines were tested for drug resistance.

#### 2.1.2 Exosome extraction

Supernatant exosomes were extracted from APSC-1 cells and the particles obtained from supernatants of untreated APSC-1 cells and gemcitabine-resistant APSC-1 cells were analyzed using nanoparticle tracking analysis.

#### 2.1.3 Cell viability assay

Treated cells were added to CCK-8 and incubated at 37°C for 4 h afterwards. The absorbance value OD450 of each well was measured using an enzyme marker.

#### 2.1.4 *In vitro* tubule formation assay

HUVEC cells were treated with each group of APSC-1 cell exosomes and divided into five groups for culture: HUVEC, HUVEC+ APSC-1-Exo, HUVEC+Ge-APSC-1-Exo, HUVEC+ APSC-1-R-Exo, and HUVEC+Ge-APSC-1-R-Exo. The above groups were assayed using CCK-8 cell activity. *In vitro* tubule formation assay was performed on five groups of HUVEC cells treated with exosomes.

#### 2.1.5 Overexpression of PPP5C construct

HUVEC and APSC-1 were overexpressed and divided into: HUVEC+control plasmid, HUVEC+PPP5C overexpression plasmid; APSC-1+control plasmid, APSC-1+PPP5C overexpression plasmid.

#### 2.1.6 WB

To detect the protein expression level of PPP5C using WB, the protein was first extracted from the cells or exosomes, followed by electrophoresis operation. After electrophoresis, a formal immunoblotting operation is performed to obtain the relevant film. The relevant primer sequences are shown in **Table 1**.

**Table 1:** Primer sequence list.

Gene	Primer	Sequence (5’-3’)
PPP5C	Forward	5’-AAGTTCTACAGCCAGGCCAT-3’
	Reverse	5’-ATCCTTGTCATGGGGCTTCA-3’
GAPDH	Forward	5’-TCAAGAAGGTGGTGAAGCAGG-3
	Reverse	5’-TCAAAGGTGGAGGAGTGGGT-3’

#### 2.1.7 QPCR

The differentially expressed miRNAs, hsa-miR-520a-5p, hsa-miR-7-1-3p, and hsa-miR-874-3p, were screened by whole-transcriptome sequencing. qPCR was then used to detect the results of these miRNAs in APSC-1 and its exosomes, and those with consistent expression changes in cells and exosomes, and those with sequencing results, were selected as indicators for subsequent studies. The RNA expression level of PPP5C, which also needs to be detected by qPCR, was selected as an indicator for subsequent studies. Relevant primer sequences. **Table 2**

**Table 2:** Primer sequences used for gene detection.

Gene	Primer	Sequence (5’-3’)
U6	Forward	CGCTTCGGCAGCACATATAC
	Reverse	AAATATGGAACGCTTCACGA
hsa-miR-520a-5p	loop primer	GTCGTATCCAGTGCAGGGTCCGAGGTATTCGCACTGGATACGACAGAAAGTA
	Forward	TGCGCCTCCAGAGGGAAGTAC
hsa-miR-7-1-3p	loop primer	GTCGTATCCAGTGCAGGGTCCGAGGTATTCGCACTGGATACGACTATGGCAG
	Forward	TGCGCCAACAAATCACAGTCTG
hsa-miR-874-3p	loop primer	GTCGTATCCAGTGCAGGGTCCGAGGTATTCGCACTGGATACGACTCGGTCCC
	Forward	TGCGCCTGCCCTGGCCCGAGGG

#### 2.1.8 Firefly luciferase

After performing cell culture and transfection using the kit, firefly and sea kidney luciferase activities were analyzed using a dual luciferase reporter assay (Promega) to verify the correspondence between hsa-miR-520a-5p and its target gene PPP5C (Luciferase Reporter assays).

#### 2.1.9 Autophagic flow assay

After overexpression treatment of HUVEC and APSC-1, the expression levels of LC3 II/I, p62 in them were detected using WB. Also for the treated cells, 2 μL of m RFP- GFP-LC3 adenovirus transfection was added to each group for 24 to 36 h. The corresponding stimuli were added to intervene for 12 h, respectively, and sent to the confocal microscope room for observation and photographic preservation to detect the cellular autophagy level.

#### 2.1.10 Bioinformatics analysis

The effect of PPP5C on the survival prognosis of pancreatic cancer was analyzed using the TCGA database.

## 3 Results

### 3.1 PPP5C overexpression increases pancreatic cancer cell activity

**Figure 1:**
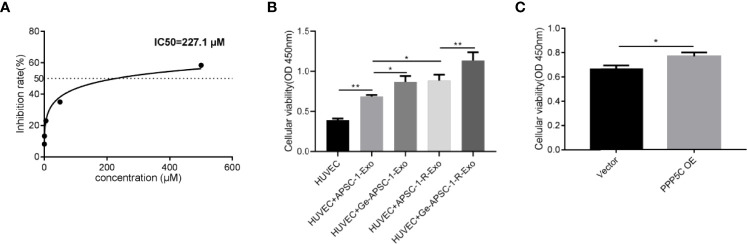
Drug resistance and cellular activity: **(A)** IC50 values of gemcitabine in ASPC-1 cells; **(B)** CCK8 detection of cell proliferation; **(C)** CCK8 detection of cell proliferation after overexpression treatment.

After CCK-8 assay, the IC50 value of gemcitabine in ASPC-1 cells was found to be 227.1 μM (**Figure 1A**). After exosome treatment, HUVEC cell viability was increased in the ASPC-1-Exo-treated group compared with the control HUVEC cells; cell viability was further upregulated in the Ge-ASPC-1-Exo-treated group and ASPC-1-R-Exo-treated group compared with the ASPC-1-Exo-treated group; Ge-ASPC-1-R-Exo-treated group was further upregulated compared with the ASPC-1-Exo-treated group; and Ge-ASPC-1-Exo-R-Exo-treated group was further upregulated compared with the ASPC-1-Exo-treated group. The cell viability was further increased in the Ge-ASPC-1-R-Exo treated group compared with the ASPC-1-R-Exo treated group (**Figure 1B**). After overexpression treatment, cell activity was increased after PPP5C overexpression treatment compared to the control group (**Figure 1C**).

### 3.2 Elevated expression of PPP5C promotes tubule formation *in vitro*

**Figure 2:**
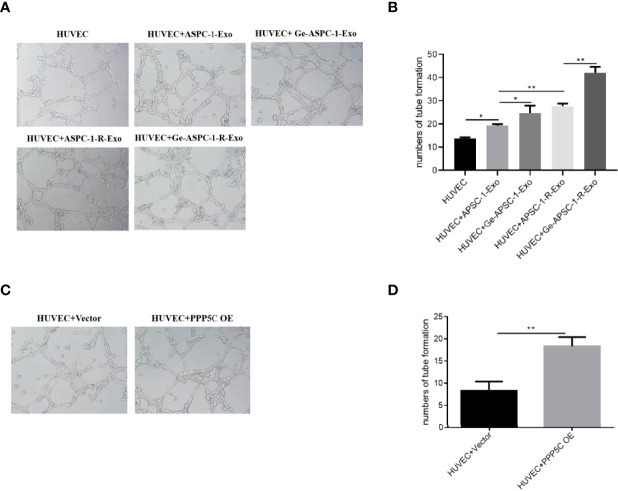
Tubule formation capacity assay **(A)** *in vitro* tubule formation capacity assay for each group (experimental plots); **(B)** *in vitro* tubule formation capacity assay for each group (bar graph); **(C)** *in vitro* tubule formation capacity assay for each group after overexpression treatment (experimental plots); **(D)** *in vitro* tubule formation capacity assay for each group after overexpression treatment (bar graph). Note: *p < 0.05, **p < 0.01.

After examination, compared with control HUVEC cells, *in vitro* tubule formation was increased in HUVEC+ASPC-1-Exo group; compared with HUVEC+ASPC-1-Exo, *in vitro* tubule formation was further increased in HUVEC+Ge- ASPC-1-Exo group and HUVEC+ASPC-1-R-Exo treated group; HUVEC+Ge- ASPC-1-R-Exo group further increased *in vitro* tubule formation compared with HUVEC+ASPC-1-R-Exo treatment (**Figures 2A, B**). And after overexpression treatment, *in vitro* tubule formation was increased after PPP5C overexpression treatment compared to the control group (**Figures 2C, D**).

### 3.3 Effect of cellular autophagy level

**Figure 3:**
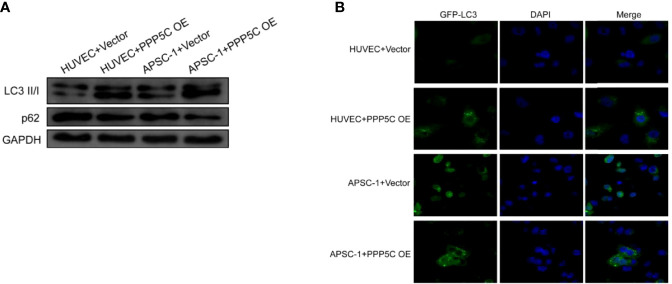
Autophagy levels: **(A)** WB autophagy level assay in each group of cells; **(B)** immunofluorescence autophagy level assay in each group of cells. .

From WB experiments, it was found that LC3 II/I expression was up-regulated and p62 expression was down-regulated in the overexpression PPP5C-treated group compared with the control HUVEC cells, and LC3 II/I expression was up-regulated and p62 expression was down-regulated in the overexpression PPP5C-treated group compared with the control APSC-1 cells (**Figure 3A**). From the immunofluorescence autophagy level, it was found that the autophagic puncta were increased in the overexpression PPP5C-treated group compared with the control HUVEC cells, and the autophagic puncta were increased in the overexpression PPP5C-treated group compared with the control APSC-1 cells (**Figure 3B**).

### 3.4 Gemcitabine treatment increased the expression of PPP5C

**Figure 4:**
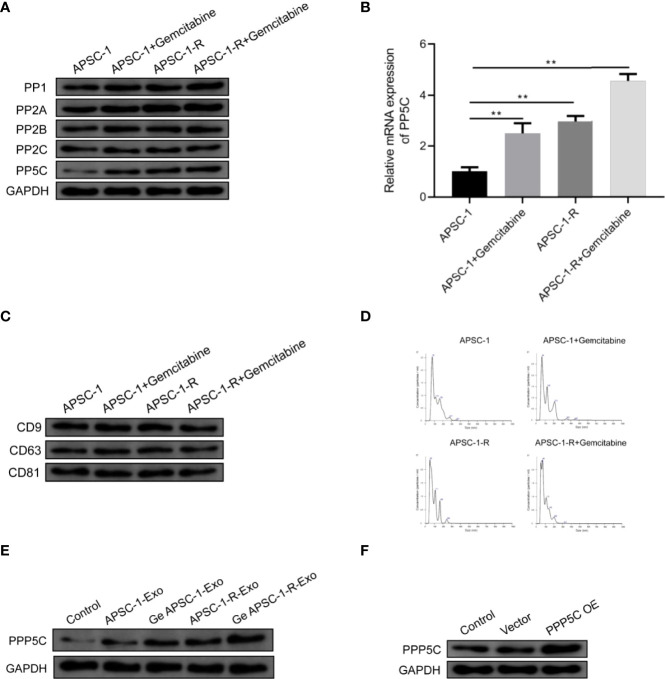
Expression of PPP5C as well as exosomal proteins: **(A)** serine/threonine protein phosphatase expression assay; **(B)** PP5C mRNA content in each group of ASPC-1 cells; **(C)** exosome markers in each group; **(D)** exosome diameter distribution in each group of cells; **(E)** PPP5C expression in each group of ASPC-1 exosomes; (F) PPP5C in each group after overexpression.

After WB as well as qPCR assays, it was found that only PP5C (i.e., PPP5C) expression was increased in the gemcitabine-treated group and in the resistant cell line compared with the control ASPC-1 cells, and PP5C was further upregulated in the resistant cell line treated with gemcitabine, while mRNA expression was also upregulated (**Figures 4A, B**). Exosomes CD9, CD63 and CD81 were significantly expressed in all groups (**Figure 4C**). Among the exosomes, the results showed that the particle size of the highest concentration in the supernatant of the ASPC-1 group was 76 nm, the average particle size of the cellular exosomes was 124.2 nm, and the concentration was 1.67×109 particles/mL; the particle size of the highest concentration in the supernatant of the ASPC-1+ Gemcitabine group was 64 nm, the average particle size of the cellular exosomes was 118.1 nm, and the concentration was 1.67×109 particles/mL. The highest particle size in the supernatant of ASPC-1-R cells was 50 nm, the average particle size of exosomes was 91.1 nm, and the concentration was 1.42×109 particles/mL; the highest particle size in the supernatant of ASPC-1-R+ Gemcitabine group was 69 nm, and the concentration of exosomes was 1.42×109 particles/mL. The highest particle size was 69 nm, and the average particle size of cellular exosomes was 93.6 nm and the concentration was 1.53×109 particles/mL (**Figure 4D**). After exosome treatment, PPP5C expression increased in the ASPC-1-Exo-treated group compared with the control ASPC-1 cells; PPP5C expression further increased in the Ge-ASPC-1-Exo-treated and ASPC-1-R-Exo-treated groups compared with the ASPC-1-Exo-treated group; PPP5C expression increased in the Ge-ASPC-1-R-Exo-treated group compared with the ASPC-1-Exo-treated group; PPP5C expression increased in the ASPC-1-Exo-treated group compared with the ASPC-1-Exo-treated group. PPP5C was further increased in the ASPC-1-R-Exo treated group compared to the ASPC-1-R-Exo treated group (**Figure 2E**). After overexpression treatment, PPP5C expression was significantly increased in HUVEC cells transfected with PPP5C overexpression vector compared with the control group (**Figure 2F**).

### 3.5 miR-520a targeted PPP5C inhibition

**Figure 5:**
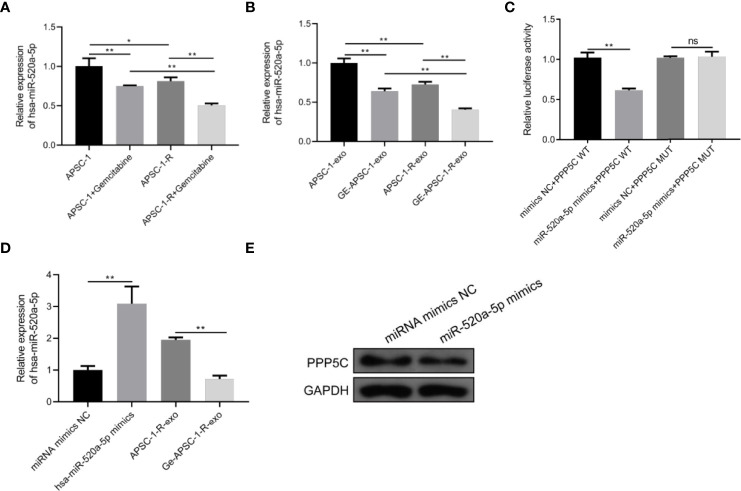
Mechanism of PPP5C expression imbalance: **(A)** hsa-miR-520a-5p expression in each group of ASPC-1 cells; **(B)** hsa-miR-520a-5p expression in each group of ASPC-1 exosomes; **(C)** luciferase activity assay; **(D)** hsa-miR-520a-5p overexpression versus hsa-miR-520a-5p in the exosomes of drug-resistant cells. miR-520a-5p expression assay; **(E)** expression level of PPP5C after hsa-miR-520a-5p overexpression. Note: * indicates p < 0.05, *p < 0.05, **p < 0.01.

Compared with control ASPC-1 cells and their exosomes, hsa-miR-520a-5p expression was decreased both in gemcitabine-administered treated and resistant cells and in treated exosomes (**Figures 5A, B**). Dual luciferase assays showed that hsa-miR-520a-5p was targeted to PPP5C (**Figure 5C**). In contrast, among HUVEC cells, hsa-miR-520a-5p expression was significantly upregulated after hsa-miR-520a-5p mimics treatment compared to control HUVEC, while PPP5C expression was significantly decreased, while compared to the exosome-treated group of resistant cells, exosome treatment after drug administration caused hsa-miR-520a-5p expression decreased. (**Figures 5D, E**).

### 3.6 Elevated PPP5C expression levels make tumor patients have worse prognosis

**Figure 6:**
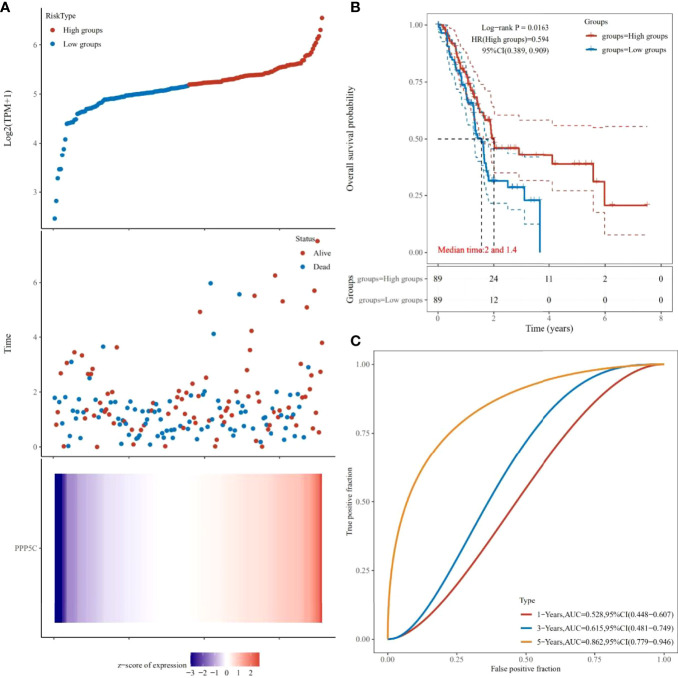
Prognostic analysis of PPP5C in pancreatic cancer patients from TCGA database: **(A)** Distribution of high and low risk samples. **(B)** Survival curves. **(C)** ROC curves.

After analysis of the TCGA database, it was found that the higher the expression level of PPP5C among tumors, the worse the prognosis of tumor patients (**Figures 6A, B**), and the ROC curve analysis revealed that PPP5C could be used more accurately to predict the survival rate of patients within 3-5 years (**Figure 6C**).

## 4 Discussion

In recent years, research on the molecular target therapy of tumors has gradually come to the forefront. In contrast, PPP5C mentioned in this study is a cancer-promoting factor in many cancers (20, 21). It has been less studied in pancreatic cancer and is only known to make tumors resistant to gemcitabine (16). In this section, we would analyze our results through the lens of relevant literature in order to find the immune-related genes of colon cancer, establish a prognostic risk score model, and verify its accuracy.

Members of the Ser/Thr protein phosphatase gene family, such as PPP1c, PPP2c, and PPP3c, play an important role in various cancers (22). If PPP5C is dephosphorylated at the P site, its highly conserved catalytic core and bimetallic system (M1/M2) will change to substrate-binding and hydrolysis sites. This will lead to the overexpression of PPP5C, which is one of the reasons for cell proliferation and the progression of various cancers (23). As an essential regulator of hormone and stress-related signal transduction (24), inhibition of its activity or expression will cause cell cycle arrest, hinder mitosis, and eventually lead to apoptosis (25). In this study, through preliminary analysis of the TCGA database, we found no significant difference in the expression of PPP5C between pancreatic cancer and normal tissues. However, the prognosis analysis showed that the higher the PPP5C level, the lower the five-year survival rate and the worse the patient’s prognosis. So, how to study the role played by PPP5C in pancreatic cancer? In a study on tumor brain metastasis, it was found that biomarker discovery was facilitated by raw letter analysis of available case information combined with clinical trials of tumor tissue (26). Another study on the expression characteristics and clinical significance of ubiquitin-specific proteases (USPs) in hepatocellular carcinoma (HCC) identified USPs as one of the eight signature genes in hepatocellular carcinoma and a potential molecular target for HCC development and progression by using raw signal analysis combined with WB and immunohistochemistry (27). With reference to the methodology used in this study, we performed a related experiment on PPP5C. In the experiments related to AsPC-1 cells, it was found that the protein and mRNA of PP5c in the gemcitabine treatment group and drug-resistant cell lines were upregulated, while in the control AsPC-1 cells and in the exosomes, the expression level of PPP5C was the same. After overexpression, with the increase of PPP5C expression level in the cell experiment, the cell activity of PPP5C increased, and the increment speed accelerated. In the tumor microenvironment, exosomes, as well as other extracellular vesicles and cytokines, act as intercellular communication agents in various biological activities that contribute to drug resistance in cancer cells and are potential targets and candidate biomarkers of drug efficacy for reversing chemoresistance in HCC patients in many cancers (28). According to Zhu etal. (16), the inhibition of PPP5C increases the expression of related apoptotic markers, indicating the apoptosis of corresponding pancreatic cancer cells. This may enhance the sensitivity of PC cells to gemcitabine. This is similar to this study.

We also conducted an experiment on the effect of PPP5C on the angiogenesis of cancer cells and autophagy. The results show that the increased expression of PPP5C promotes angiogenesis and autophagy. After the tumor develops to an advanced stage, autophagy, as a dynamic degradation and circulatory process, contributes to the survival and growth of established tumors and can effectively promote metastasis and cancer invasiveness (29). Combined with our results on autophagy, we can conclude that PPP5C can promote the metastasis of pancreatic cancer cells. Furthermore, our angiogenesis experiments suggest that PPP5C can promote further deterioration of the tumor. This is sufficient to confirm the prognostic analysis results that the higher the level of PPP5C, the lower the survival rate within five years and the worse the prognosis of patients. In this connection, our study effectively proves that the role of PPP5C in pancreatic cancer is similar to that in other cancers.

At the same time, compared with previous studies on PPP5C and pancreatic cancer, this study examined the expression of miR-520a-5p in pancreatic cancer and its relationship with PPP5C. Although miR-520a-5p exists as a pathogenic factor in psoriasis and cardiomyocyte injury, studies on various human cancers, such as colorectal cancer and hepatocellular carcinoma, have found that miR-520a-5p inhibits tumor development (30). In this context, let us look at the results of the miRNA experiment in this study. Although the databases we used do not support the relationship between miR-520a-5p and PPP5C, there is a lack of relevant literature. From the results of the double luciferase experiment in this study, miR-520a had a targeted inhibitory effect on PPP5C after overexpression. At the same time, we also found that its expression decreased in drug-resistant strains. From these results, we can conclude that miR-520a-5p can inhibit the growth of pancreatic cancer by targeting PPP5C inhibition.

In conclusion, this study explored the role of PPP5C in pancreatic cancer by combining bioinformatics analysis and experiments and found the target relationship between miR-520-5p and PPP5C. This provides a new molecular mechanism for pancreatic cancer research and offers new directions for targeted gene therapy.

## Data Availability Statement

The original contributions presented in the study are included in the article/supplementary material. Further inquiries can be directed to the corresponding author/s.

## Author Contributions

All authors contributed to the article and approved the submitted version.

## Funding

This work was supported by Zhejiang Province Bureau of Health under grant 2020366835 and Funds of Science Technology Department of Zhejiang Province under grant 2020C03074.

## Conflict of Interest

The authors declare that the research was conducted in the absence of any commercial or financial relationships that could be construed as a potential conflict of interest.

## Publisher’s Note

All claims expressed in this article are solely those of the authors and do not necessarily represent those of their affiliated organizations, or those of the publisher, the editors and the reviewers. Any product that may be evaluated in this article, or claim that may be made by its manufacturer, is not guaranteed or endorsed by the publisher.
